# IgG Seroreactivites to Viral Capsid Protein VP1 of JC and BK Polyomaviruses in Children at Early Ages with Special Reference to Parental Cofactors

**DOI:** 10.3390/children10101645

**Published:** 2023-10-01

**Authors:** Hanna K. Laine, Tim Waterboer, Kari Syrjänen, Seija Grenman, Karolina Louvanto, Stina Syrjänen

**Affiliations:** 1 Department of Oral and Maxillofacial Diseases, University of Helsinki, 00290 Helsinki, Finland; 2 Department of Oral Pathology and Radiology, Faculty of Medicine, University of Turku, 20520 Turku, Finland; stina.syrjanen@utu.fi; 3 Division of Infections and Cancer Epidemiology, German Cancer Research Center (DKFZ), 69120 Heidelberg, Germany; t.waterboer@dkfz.de; 4 SMW Consultants, Ltd., 21620 Kaarina, Finland; kasyrja@saunalahti.fi; 5 Department of Obstetrics and Gynecology, University of Turku, Turku University Hospital, 20014 Turku, Finland; seija.grenman@tyks.fi; 6 Department of Obstetrics and Gynecology, Faculty of Medicine and Health Technology, Tampere University, 33520 Tampere, Finland; karolina.louvanto@tuni.fi; 7 Department of Obstetrics and Gynecology, Tampere University Hospital, 33100 Tampere, Finland; 8 Department of Pathology, University of Turku, Turku University Hospital, 20520 Turku, Finland

**Keywords:** BK polyomavirus, JC polyomavirus, infant, mother, father, seroprevalence, seroconversion

## Abstract

BK (BKPyV) and JC (JCPyV) polyomaviruses are widespread in humans. Transmission at an early age and the role of parents in spreading these viruses through the family are incompletely understood. Our aim was to determine the seroprevalence of BKPyV and JCPyV in infants at the age of 1, 2, 6, 12, 24, and 36 months and to assess the frequency of BKPyV and JCPyV seroconversion. A variety of maternal and paternal covariates were also tested as potential predictors of these early childhood infections. We used multiplex serology to analyze antibodies to BKPyV and JCPyV from baseline to 3-year follow-up visits. We observed that there was nearly perfect correlation in BKPyV and JCPyV serum IgG antibody levels between the mother-infant pairs during the first year of the infant’s life. No correlation among BKPyV antibody titers were found in father–child pairs, whereas JCPyV antibody levels of the father and child had a significant correlation at the 2-year follow-up visit. BKPyV infection may be associated with a child’s predisposition to allergy. In conclusion, after the decay of maternal antibodies, children start to develop their own immunity toward BKPyV and JCPyV, and horizontal transmission of infection in the family can occur.

## 1. Introduction

The human polyomavirus (HPyV) family comprises 13 members, of which BK (BKPyV) and JC (JCPyV) polyomaviruses were the first two to be recognized [1,2]. BKPyV and JCPyV infections are common and life-long in the general population and usually do not have any serious consequences [3]. The seroprevalence of BKPyV and JCPyV in adults is 90% and 50–70%, respectively [4,5,6,7,8,9]. However, among immunocompromised individuals, e.g., renal transplant recipients, BKPyV and JCPyV can cause polyomavirus-associated nephropathy and multifocal leukoencephalopathy, respectively.

The presence of maternal antibodies to BKPyV and JCPyV in newborns is well known. However, there are nearly no longitudinal studies on BKPyV and JCPyV serology in infants. There are limited studies on the time of seroconversion to BKPyV and JCPyV. Studies on the correlation of BKPyV and JCPyV antibody titers in the offspring with those of the parents are also lacking. In particular, data on the paternal effect of offspring serostatus are lacking. In a study from Italy, Elia and coworkers suggested that primary infection with JCPyV occurs during the first 6 months of life, and children become seropositive before the age of 2 years [10]. A Swedish study by Stolt and coworkers showed that nearly 98% of children at the age of 7–9 years were BKPyV seropositive, whereas JCPyV seroprevalence in the same age group was only 51% [6]. The few available longitudinal studies suggest that serum IgG antibody levels to the virus-like particles (VPL) BKPyV and JCPyV are stable over time, and any changes in seroprevalence can be used as an indicator of cumulative exposure to these viruses [11]. Boldorini et al. analyzed 19 mother–newborn pairs and confirmed that maternal BKPyV and JCPyV IgG antibodies were present in the newborns [12]. The Rhea birth cohort study from Greece showed that seroprevalence to BKPyV in cord blood was nearly two-fold higher than that of JCPyV (96% vs. 53%). At the age of 4 years, 73% and 30% of the offspring were seropositive to BKPyV and JCPyV, respectively [13].

Given that longitudinal studies in early infancy are nearly nonexistent, we analyzed BKPyV and JCPyV serology among children included in the Finnish Family Human Papillomavirus (FFHPV) Study cohort who were followed up for the first 3 years of life. Our aim was to determine the seroprevalence of BKPyV and JCPyV in infants at the ages of 1, 2, 6, 12, 24, and 36 months and the maternal association of these antibodies. The possible impact of the father on offspring BKPyV and JCPyV serology was also analyzed, although it was not possible to study the transmission modes of these viruses within the current setting of this study. A longitudinal study setting was exploited to assess the frequency of BKPyV and JCPyV seroconversion among these infants, and a variety of maternal and paternal covariates were tested as potential predictors of these early childhood infections.

## 2. Materials and Methods

### 2.1. Individuals in This Study

This study consisted of families from the FFHPV study, which is a longitudinal cohort originally designed to explore the dynamics of HPV infection in enrolled mothers, fathers, and their unborn children. Between 1998 and 2001, 327 pregnant women (in the third trimester) and their male spouses were enrolled at the Maternity Unit of Turku University Hospital (Finland). The Research Ethics Committee of Turku University Hospital (#3/1998, with amendments 45/1801/2018) approved the design of this study. All procedures were performed in accordance with relevant guidelines and regulations. Written informed consent was obtained from all participants. The demographic data were obtained from the participants using a standardized questionnaire, as reported previously [14].

### 2.2. Collection of Blood Samples

Blood samples collected during the FFHPV study were available for the present study, as described previously [15]. In brief, blood samples were collected from all 327 pregnant women and from all 132 enrolled spouses at baseline and at 12-, 24-, and 36-month follow-up (FU) visits. Results of the BKPyV and JCPyV serology analysis of the spouses were published recently [9]. In the present study, the baseline BKPyV and JCPyV serologies of the parents were included. In addition, the fathers’ BKPyV and JCPyV serology data were used as part of the analysis of father–child pairs at the 12-, 24-, and 36-month visits. Although the intended sampling schedule included blood samples from children at the 1-, 2-, 6-, 12-, 24-, and 36-month FU visits, the actual timing (mean months and range) of the FU visits were as follows: 1.2 (0.5–1.9), 2.2 (1.6–3.8), 6.4 (5.2–9.4), 12.6 (10.4–15.5), 24.8 (21.4–32.9), and 36.9 (34.1–47.5) months.

The number of blood samples for BKPyV and JCPyV serology during FU is shown in Table 1 and Table 2. Blood samples from the parents were taken according to standard procedures of the Turku University Hospital. The first blood sample for a newborn was taken from the head, while the subsequent samples were drawn from the arm bend. Serum samples were immediately divided into three 1 mL aliquots and stored first at −20 °C (max. 1 week) and then at −70 °C. In total, 100 μL of sera were shipped on dry ice to the German Cancer Research Center (DKFZ), Heidelberg (Germany), for serological analysis.

### 2.3. Fluorescent Bead-Based Multiplex Serology

Serum IgG antibodies to major capsid protein VP1 of BKPyV and JCPyV were measured using fluorescent bead-based multiplex serology [16,17]. The BKPyV and JCPyV serology assay was described in detailed previously by Antonsson et al. and Gossai et al. [7,8]. In the present study, the cutoff value of the BKPyV and JCPyV seropositivity was ≥400 MFI (VP1 antigen-specific mean fluorescence intensity), which was also used in a previous study by Antonsson et al. (BKPyV and JCPyV serology was performed in the same laboratory at the German Cancer Research Center, Heidelberg, Germany) and also by Carter et al. [7,18].

Seroconversion was defined by the following two conditions, both of which must be fulfilled: at least a two-fold increase in the previous serum MFI value and an MFI value increasing from below to above the 400 MFI cutoff. Similarly, antibody waning (decay) was defined by the following two conditions: at least a two-fold decrease in the previous serum value and a decrease in the MFI value from above to below the 400 MFI cutoff. The demographic data recorded using the detailed questionnaires at study entry were used to identify potential predictors of BKPyV and JCPyV seroconversion among the offspring.

### 2.4. Statistical Analysis

All statistical analyses were performed with SPSS for Windows (version 28.0.1.1; SPSS, IBM Corp., Armonk, NY, USA) statistical software. The bivariate correlation between the BKPyV and JCPyV antibody levels between maternal sera (baseline) and her offspring’s sera taken at the 1-, 2-, 6-, 12-, 24-, and 36-month visits was tested using Spearman r correlation coefficients separately for both polyomaviruses. The same test was also used to calculate the correlations in the father–offspring pairs at different time points. Conventional 2 × 2 tables were used to analyze categorical variables, which were tested using the likelihood-ratio test or Fisher–Freeman–Halton exact test. Differences in the means of continuous variables were analyzed using ANOVA or a Mann–Whitney/Kruskal–Wallis test for two and multiple independent samples, respectively. All statistical tests were performed two-sided and were considered significant when *p* < 0.05.

## 3. Results

### 3.1. Demographic and Clinical Data on Mothers, Fathers, and Their Offspring at Birth

The mean age of the mothers was 25.5 years (range, 18–38 years). Of the 327 mothers, 44% were current or past smokers, and 84% started smoking before the age of 17 years. Over half (56%) had their sexual debut between 14 and 16 years of age. Of the mothers, 22% had only one to two lifetime sexual partners, and 60 mothers (18%) reported >10 sexual partners. Among users of oral contraceptives (OC), 41% had initiated OC use between 14 and 16 years of age; 7% had never used OC. Altogether, 24% of the mothers reported a history of sexually transmitted infection (STI).

The mean age of the fathers was 29 years (range, 19–64 years). Of the fathers, 63% (75/119) had never smoked. Of the 49 smokers, 60% started smoking at the age of 14–17 years. Most of the fathers (87%) reported having ≥three sexual partners, and over half reported a history of an STI.

Of the 329 newborns (2/327 mothers delivered twins), 22% were delivered by cesarean section, and 58% were firstborn. The median birth weight was 3580 g (range, 1750–4950 g), and the mean time from membrane rupture to starting delivery was 4 h (range, 0–53 h). A total of 155 (48%) were boys and 172 (52%) were girls (two were missing data).

### 3.2. BKPyV and JCPyV Seroprevalences in the Families

#### 3.2.1. BKPyV and JCPyV Seroprevalence of Parents at Baseline and during the Follow-Up

Table 1 summarizes the seroprevalence of BKPyV and JCPyV for the parents at the baseline visit when the women were in the third trimester of pregnancy. In both mothers (95%) and fathers (96%), BKPyV seropositivity was more common than JCPyV seropositivity (58% and 66%, respectively).

#### 3.2.2. BKPyV and JCPyV Seroprevalence of Offspring at Baseline and during the Follow-Up

BKPyV seroprevalence among the offspring was highest at the 1-month visit (92.2%), followed by a decline to 7.7% at the 12-month visit, and a new increase up to 55.6% at the 36-month visit (Table 1). The longitudinal pattern of JCPyV seroprevalence was similar, but the rates were always lower than those for BKPyV. At the age of 1 month, 52.6% of the infants were JCPyV seropositive, followed by a sharp decline at the 6-month visit (4.6%). JCPyV seroprevalence was rarest (2.9%) at the age of 12 months, after which it increased to 32.5% by the age of 3 years.

### 3.3. BKPyV and JCPyV Antibody Levels (Mean MFI) of the Children during the First 36 Months of Life

**Figure 1:**
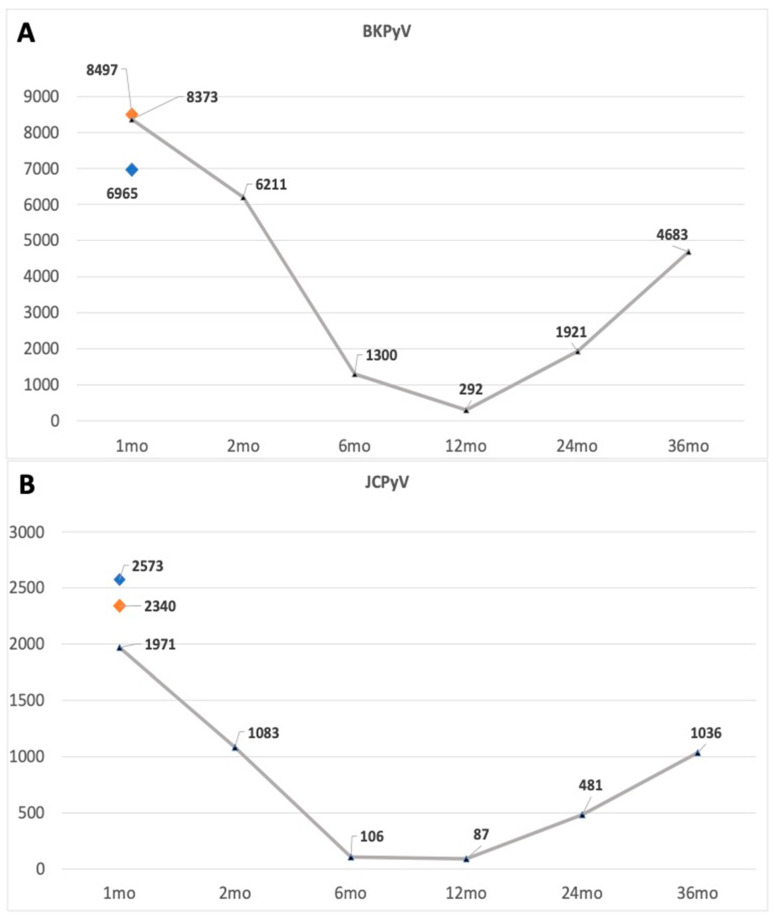
Antibody titers of BKPyV (**A**) and JCPyV (**B**) during the first 36 months of life of the children (gray lines), shown as mean MFI values. Orange dots show the mothers’ corresponding antibody titers at the baseline visit. Blue dots indicate the baseline antibody titers of the father.

Figure 1 summarizes the BKPyV and JCPyV antibody levels of the children during the first 36 months of life (line graphics) and those of the mothers at baseline (orange dots). Among newborns, BKPyV antibody levels were clearly higher than JCPyV titers at the 1-month visit (8373, BKPyV; 1971, JCPyV). These values closely paralleled the mothers’ MFI values at baseline (8497 and 2340 for BKPyV and JCPyV, respectively). At the 6-month visit, the infants’ mean MFI of BKPyV (1300) was still above the 400 MFI cutoff, whereas the mean values for JCPyV (106) were below the MFI cutoff. At the 24- and 36-month visits, both BKPyV and JCPyV titers substantially increased, and BKPyV titers were over four-fold greater than those of JCPyV.

#### 3.3.1. Correlation of the Mother’s Baseline BKPyV and JCPyV Antibody Levels to Those of Their Infants during the Follow Up

Table 2 summarizes the correlation between the mother’s baseline BKPyV and JCPyV antibody levels (pregnant at third trimester) to those of their infants during the 36-month FU. The correlation between the mother’s baseline BKPyV titers and that of her offspring was statistically significant at the 1-month, 2-month, 6-month, and 12-month visits (Spearman’s test *p* < 0.001 for all). The significant correlation between mother–offspring pairs was lost at the 24- and 36-month visits. Similarly, baseline JCPyV antibody levels of the mother were significantly correlated with the levels of the offspring at the 1-month, 2-month, and 6-month visits (*p* < 0.001 for all) and at the 12-month visit (*p* = 0.024). This significant correlation was lost at the 24- and 36-month visits (Table 2).

#### 3.3.2. Antibody Correlations in Father–Child Pairs

The correlation between BKPyV antibody titers of the fathers and their children was determined at the following four time points (Table 2): father’s baseline (before the birth of the child) titers versus that of the infant at the age of 1 month; 12-month visit for both; 24-month visit for both; and 36-month visit for both. No correlations were found between the fathers’ BKPyV antibody titers and the corresponding titers of the children at any of these four time points (*p* = 0.958, *p* = 0.996, *p* = 0.660, and *p* = 0.776, respectively). The same analyses were repeated for the JCPyV antibody titers of fathers and their children (Table 2). No correlations were found at the baseline and 12-month visits (*p* = 0.709 and *p* = 0.504, respectively). Interestingly, at the 24-month visit, JCPyV antibody titers of the fathers and their children were significantly correlated (Spearman’s test, *p* = 0.002), but this correlation disappeared by the age of 3 years (*p* = 0.162).

### 3.4. BKPyV and JCPyV Seroconversion among Infants and Its Correlation with Seroconversion of Parents

#### 3.4.1. BKPyV and JCPyV Seroconversion among Infants and Its Correlation with Mothers

We next assessed whether any correlation existed between the infants and their parents for seroconversion to BKPyV and JCPyV. Of the children, 34 (11.0%) and 29 (6.3%) seroconverted to BKPyV and JCPyV, respectively. When the effect of maternal BKPyV or JCPyV antibodies was excluded (i.e., after the waning of these antibodies), there was no correlation between a seroconverted mother and her child (*p* = 0.741 and 0.497, respectively). Forty-eight mothers had seroconverted to BKPyV, but only four (8.3%) of their children were seroconverted. In contrast, BKPyV seropositivity remained stable in 245 mothers without any significant increases in antibody levels, whereas 30 of their children seroconverted to BKPyV. Similarly, BKPyV antibody decay was detected in 17 mothers but in only two (11.8%) of their children. In total, although 58 mothers seroconverted to JCPyV during the 3-year FU, only three of their children were seroconverted. JCPyV seropositivity remained stable during the FU in 235 mothers, whereas 21 children seroconverted to JCPyV. JCPyV antibodies waned in 16 mothers, and this antibody decay was found only in one of their children.

#### 3.4.2. BKPyV and JCPyV Seroconversion among Infants and Its Correlation with Fathers

There was no significant correlation between the seroconversion of fathers and their children to BKPyV (*p* = 0.295). Altogether, 17 fathers seroconverted to BKPyV, but only two (11.8%) of their children were seroconverted. BKPyV serology remained stable in 113 fathers, whereas 12 of their children seroconverted to BKPyV. There was no association between fathers and their children for JCPyV (*p* = 0.730). In total, 25 fathers and only two (8.0%) children seroconverted to JCPyV. Of the 107 fathers who did not seroconvert at FU (they could have been seropositive for the whole period), 11 (10.3%) of their children seroconverted to JCPyV. Thus, the father’s seroconversion did not explain the child’s seroconversion.

### 3.5. Predictors of BKPyV and JCPyV Infection at an Early Age

The demographic data originally collected for the FFHPV study were used to determine the possible covariates for BKPyV and JCPyV seropositivity. As BKPyV and JCPyV seropositivity is almost ubiquitous among these young children, with antibody levels showing a wide range, more stringent cut-offs (MFI ≥ 1000) for BKPyV and JCPyV seropositivity were used in these analyses to identify the genuine risk factors that could explain BKPyV or JCPyV infections (or both) at this early age.

The results for the selected covariates are listed in Table 3. The only factor that was associated with high (MFI ≥ 1000) BKPyV antibody levels at the age of 2 and 3 years was a history of allergy (*p* = 0.037), which increased the risk of high-titer BKPyV infection (odds ratio (OR) = 1.47). The mode of delivery, breastfeeding, passive cigarette smoking, atopy, and other disorders had no impact on BKPyV. None of the examined covariates had any association with JCPyV seropositivity at this high-titer cutoff.

### 3.6. Infants Permanently Negative for BKPyV and JCPyV Antibodies Regardless of Maternal Antibody Status

We also identified children who did not have maternal antibodies to BKPyV or JCPyV regardless of the maternal BKPyV and JCPyV antibody status. There were 18 infants who were considered seronegative to BKPyV (range, 28–319 MFI, mean: 183 MFI) and 110 infants who were seronegative to JCPyV (range, 1–329 MFI, mean: 107 MFI) 1 month after delivery. Only one of these infants (323 MFI) was near the MFI 400 cutoff.

### 3.7. Family Members Permanently Negative for BKPyV and JCPyV Antibodies

Among the FFHPV cohort families, some were permanently negative to BKPyV and JCPyV. Among the mothers, two (range, 69–385 MFI) and 51 (range, 0–385 MFI) were negative to BKPyV and JCPyV, respectively, during the entire FU of 36 months. Among the fathers, two (range, 31–235 MFI) and 14 (range, 2–377 MFI) were seronegative to BKPyV and JCPyV, respectively, during the entire follow-up. Altogether, there were three constantly BKPyV seronegative children (range, 0–319 MFI); their parents were not seronegative to BKPyV. Of the 49 JCPyV seronegative children (range, 0–329 MFI), four of their fathers and 22 of their mothers were also JCPyV seronegative. However, there was not a single family where all studied family members (parents and offspring) were BKPyV and JCPyV seronegative.

## 4. Discussion

We observed that there was nearly perfect correlation in BKPyV and JCPyV serum IgG antibody levels between the mother-infant pairs during the first year of the infant’s life. In contrast, no correlation among BKPyV antibody titers was found in the father–child pairs, whereas JCPyV antibody levels of the father and the child had a significant correlation at the 2-year FU visit.

Similar to our findings, Boldorini and coworkers showed that maternal BKPyV and JCPyV IgG antibodies were detectable in newborns [12]. However, only 19 mother–infant pairs were studied, and the FU was only 1 month after delivery [12]. Interestingly, a few infants at the age of 1 month became transiently IgM seropositive, indicating a maternally transmitted infection during pregnancy or even during the neonatal period [12]. In our study, we analyzed only IgG antibodies. In contrast to our observations and those of Boldorini, Elia et al. have suggested that primary infection with JCPyV could occur during the first 6 months of life [10]. However, our results indicate that infants at the age of 6 months still have antibodies of maternal origin. Maternal antibodies to BKPyV and JCPyV IgG decayed over the first year of life, as evidenced by the lowest antibody levels in the offspring at the 12-month visit. After this time point, an infant’s own IgG synthesis is well established [19,20]. Earlier studies have highlighted the possible transmission of JCPyV within the family [13]. For this reason, we also analyzed the impact of the father on the offspring’s elevated BKPyV and JCPyV antibody levels from the age of 1 year onward. No correlation was found among BKPyV antibody titers in the father–child pairs. However, there was a correlation between JCPyV antibody levels of the father and the child that was only observed at the 24-month visit. We can speculate that mothers participate more in the child’s early life and fathers start to contribute more when the child is a toddler. The correlation between JCPyV antibody titers in the father and child pairs at the 2-year visit is a new observation and has not been presented previously. Importantly, we cannot trace the origin of these infections in children within the current setting of this study.

Human polyomavirus antibodies in early life have been studied in the Rhea birth cohort in Greece [13]. We can compare our results with this study because both were analyzed with the same antibody assay [13]. However, it is worth noting that we used a higher cutoff for BKPyV and JCPyV seropositivity (MFI 400 instead of 250 MFI). We found a lower BKPyV seroprevalence in children at age 3 years than that reported in the Greek study (20% vs. 38%). We can speculate that our more stringent cutoff and higher number of participants (243 vs. 81 children) had some impact on our lower seroprevalence rates. However, maternal BKPyV and JCPyV positivity rates at baseline were the same (95–96% and 58–53%, respectively) despite the fact that we analyzed the sera of pregnant women and the Greek study used cord blood samples. Our FU was terminated at the 36-month visit. In a Swedish study, BKPyV seroprevalence in children aged 7–9 years was 98%, and JCPyV seroprevalence was 51% in children aged 9–11 years [6].

In our study, the presence of an allergy was the only general health-related factor that was associated with BKPyV positivity. Karachaliou and coworkers analyzed the determinants of polyomavirus acquisition in childhood [13]. They found that lifestyle and health-related factors were associated with BKPyV seropositivity at age 4 years. Predictors included age at daycare entry and swimming pool attendance [13]. JCPyV seropositivity was only associated with JCPyV-positive cord blood at the delivery [13]. Contrary to their results, we did not find any association between the mode of delivery and BKPyV and JCPyV seropositivity. Recently, the same group reported that children seropositive to BKPyV were more likely to have eczema at age 4 and 6 years (OR = 1.08 and OR = 1.26, respectively) compared with their seronegative counterparts. Similarly, BKPyV increased the risk for rhinoconjunctivitis by 1.6- and 2.5-fold in the same age groups [21].

To our knowledge, the present study is the first to analyze the impact of mothers and fathers on their offspring’s BKPyV and JCPyV infection in early life and on their acquisition of immunity after 1 year of age. Transmission routes of polyomaviruses remain unknown. Possible routes of transmission are via the respiratory tract, the bloodstream to target organs such as the kidney and brain, ingestion of water contaminated by urine or stool, sexual transmission, transfusion of blood products, kidney transplantation, and vertical transmission from mother to child [22]. Vertical transmission has different modes, such as peri-conceptual transmission during fertilization or thereafter, prenatal transmission in utero during pregnancy, perinatal transmission during vaginal delivery, and transmission during childcare (nursing). Transplacental transmission of BKPyV and JCPyV has been studied previously [23]. Taguchi and coworkers found that BKPyV IgM antibodies were frequent in non-pregnant women, in women who experienced spontaneous abortion, and in pregnant women in the first trimester and after delivery. They also speculated that BKPyV could remain latent in healthy women and activate during pregnancy [23]. An experimental study on mice showed that transplacental transmission of polyomavirus is possible and that the virus pooled in kidney samples [24]. Later, Zhang et al. showed that (similar to murine polyomavirus infection) the viral load is highest 7 days after birth in the salivary gland, kidney, liver, and spleen [25]. Boldorini et al. studied aborted fetuses and found that BKPyV can cross the placenta and remain latent in fetal organs without increasing the risk of abortion [26]. However, they did not detect BKPyV or JCPyV in the umbilical cord blood samples of newborns, even when the mother’s urine and blood samples were HPyV positive [26]. In contrast, Mazzoni et al. found BKPyV and JCPyV DNA in 3% and 7% of umbilical cord blood samples, respectively [27]. This indicates that vertical transmission is possible. In our study, 12% and 10% of the children seroconverted to BKPyV and JCPyV, respectively. However, we cannot trace the origin of these infections as we did not assess the presence of viral DNA or RNA in the offspring or in the parents but rather assessed serology for BKPyV and JCPyV. After the waning of maternal antibodies, no statistically significant correlation was found among seroconverted mother–child pairs or father–child pairs.

We also identified children and parents who remained BKPyV and JCPyV seronegative during the entire FU. Possible explanations include tolerance to these viruses or mutations in host proteins required for viral entry, infection, or both [28]. The maternal immune system and immunomodulation during pregnancy were recently described, not only at the fetal–maternal interface but also at a systemic level. In this context, the role of mucosal-associated invariant T (MAIT) cells have been of particular interest. These cells are innate-like T cells [29].

The strength of this study is the unique family cohort of healthy subjects with a long FU. There are previous population-based studies on seroprevalences of different polyomaviruses. Kean and co-others found that 38% and 16% of 112 children aged 1–5 years were seropositive to BKPyV and JCPyV, respectively [5]. In the current study, we analyzed seroprevalences of healthy parents and their children during a 3-year FU. To our knowledge, our study is the only study that reported individual changes in the antibody titers and seroprevalence longitudinally during the FU. However, not all family members were motivated to participate in a 3-year study with several visits and samplings. This was evident from the decreasing number of individuals, thus impairing the statistical analyses.

## 5. Conclusions

IgG antibody levels of BKPyV and JCPyV in infants correlate with those of their mothers from birth to 1 year. BKPyV antibody titers between father–child pairs had no correlation. However, there was a significant correlation between father and child pairs when the child was at the age of 2 years. At the age of 3 years, half of the children had their own antibodies to BKPyV, while only 33% had antibodies to JCPyV. BKPyV antibody titers were 4-fold higher than JCPyV titers during the first 6 months of life and from the age of 2 years to the end of this study. Furthermore, BKPyV infection may be related to the propensity of allergy. In conclusion, BKPyV and JCPyV infections in early childhood are common. The transmission routes remain unclear. Close contact with family members may be one possibility.

## Figures and Tables

**Table 1:** Seroprevalence of BK (BKPyV) and JC (JCPyV) polyomaviruses of expectant parents and their offspring by follow-up.

	Mother	Father	Offspring Seroprevalence during Follow-Up over 3 Years (MFI ≥ 400 MFI)					
Follow-Up Visit	Baseline When Pregnant at Third Trimester	Baseline	1-Month	2-Month	6-Month	12-Month	24-Month	36-Month
**BKPyV**								
**seroprevalence %**	94.8	95.5	92.2	84.8	46.4	7.7	19.6	55.6
**(sero+/total N)**	(301/327)	(126/132)	(214/232)	(201/237)	(122/263)	(21/272)	(49/250)	(135/243)
**JCPyV**								
**seroprevalence %**	58.3	65.9	52.6	39.2	4.6	2.9	12.4	32.5
**(sero+/total N)**	(193/331)	(87/132)	(122/232)	(93/237)	(122/263)	(21/272)	(49/250)	(135/243)

**Table 2:** Correlation of BK (BKPyV) and JC (JCPyV) polyomaviruses antibody levels at baseline of the (when pregnant) mother with those of her offspring by follow up. Similar correlation was determined between the father-offspring pairs at a different follow-up time.

	Spearman’s Correlation Coefficient r (*p* = Significance of the Correlation)					
Follow-Up Visit	1-Month	2-Month	6-Month	12-Month	24-Month	36-Month
**Number of mother–child pairs**	***n*** ** = 231**	***n*** ** = 239**	***n*** ** = 262**	***n*** ** = 271**	***n*** ** = 249**	***n*** ** = 242**
**BKPyV**	0.928 (0.001)	0.897 (0.001)	0.842 (0.001)	0.451 (0.001)	0.077 (0.226)	0.007 (0.909)
**JCPyV**	0.965 (0.001)	0.946 (0.001)	0.781 (0.001)	0.137 (0.024)	0.087 (0.173)	0.041 (0.522)
**Number of father–child pairs**	***n*** ** = 97**			***n*** ** = 106**	***n*** ** = 77**	***n*** ** = 87**
**BKPyV**	0.005 (0.958)			0.001 (0.996)	0.051 (0.660)	0.031 (0.776)
**JCPyV**	0.038 (0.709)			0.066 (0.504)	0.340 (0.002)	0.151 (0.162)

**Table 3:** Association of children’s demographic data with BKPyV and JCPyV seropositivity.

	BKPyV+	BKPyV−	*p*-Value	JCPyV+	JCPyV−	*p*-Value
**Mode of delivery**						
Vaginal	29 (14.6)	170 (85.4)	1.000	13 (76.5)	187 (78.9)	1.000
Cesarian section	8 (14.3)	48 (85.7)		4 (23.5)	50 (21.1)	
**Breastfeeding**						
Yes	26 (70.3)	174 (79.8)	0.199	13 (76.5)	187 (78.9)	1.000
No	11 (29.7)	44 (20.2)		4 (23.5)	50 (21.1)	
**Passive cigarette smoking**						
Yes	15 (46.9)	106 (51.0)	0.707	7 (41.2)	114 (51.6)	0.458
No	17 (53.1)	102 (49.0)		10 (58.8)	107 (48.4)	
**Atopia**						
Yes	7 (21.9)	31 (15.4)	0.438	2 (13.3)	47 (27)	0.361
No	25 (78.1)	170 (84.6)		13 (86.7)	127 (73)	
**Presence of allergy**						
Yes	20 (62.5)	87 (42.4)	0.037 *	7 (41.2)	99 (45.4)	0.804
No	12 (37.5)	118 (57.6)		10 (58.8)	119 (54.6)	
**Disorders**						
Asthma	2 (6.3)	7 (4.5)	1.000	2 (13.3)	7 (4.0)	0.340
Other	0 (0.0)	3 (1.9)		0 (0.0)	3 (1.7)	
Healthy	30 (93.8)	147 (93.6)		13 (86.7)	165 (94.3)	

* (Odds ratio) OR = 1.473, (95% confidence interval 1.078–2.012).

## Data Availability

All data generated and analyzed for the current study are included in this article.
